# Hydrodistillation time-dependent variations in the volatile oil characteristics of fresh and dried Salvia species

**DOI:** 10.1038/s41598-026-42109-7

**Published:** 2026-02-27

**Authors:** Emir Soltanbeigi

**Affiliations:** Department of Medicinal and Aromatic Plants, Atatürk Health Care Vocational School, Afyonkarahisar Health Sciences University, Afyonkarahisar, Turkey

**Keywords:** Essential oil, Hydrodistillation time, S. fruticosa, S. officinalis, Biochemistry, Biological techniques, Chemical biology, Chemistry, Drug discovery, Plant sciences

## Abstract

Salvia species are valued for their medicinal and aromatic properties, which largely arise from their volatile oils (VOs). The content and chemical composition of VOs depend on factors such as harvest time, post-harvest processing, and extraction conditions. This study investigated the effects of hydrodistillation (HD) duration and leaf state (fresh vs. dried) on the content and chemical composition of VOs from *Salvia officinalis* and *Salvia fruticosa*. Leaves were distilled for 1–5 h, and VOs were analyzed using GC/FID-MS. In *S. officinalis*, VO content increased with HD time (0.37–0.64% in fresh leaves; 1.48–2.00% in dried leaves). In dried leaves, most of the recoverable VO was obtained within 3 h, with only limited additional increases thereafter, indicating diminishing returns beyond ~ 3 h under the applied conditions. In *S. fruticosa*, VO content increased in fresh leaves from 0.42% to 0.50% and in dried leaves during the first three hours (1.2–1.52%), although fresh leaves provided a higher VO content relative to the original fresh weight. GC/FID-MS identified 41 and 32 compounds in fresh and dried *S. officinalis*, and 49 and 48 in *S. fruticosa*. α-Thujone was the predominant component in both species, showing its highest relative abundance at 1 h and decreasing with prolonged HD; a similar pattern was observed for 1,8-cineole, consistent with time-dependent shifts in the volatile profile. α-Pinene was higher in dried *S. fruticosa* (6.62–9.28%) than fresh (4.62–7.45%). Oxygenated monoterpenes were the most abundant chemical group, while sesquiterpenes and diterpenoids increased with extended HD. These findings highlight species-specific extraction dynamics and underscore the importance of selecting HD duration according to the intended balance between VO content and compositional profile.

## Introduction

Volatile oils (VO) are aromatic and volatile substances obtained through extraction from various parts of plants, such as flowers, leaves, roots, bark, fruits, and seeds^1^. They possess diverse biological activities (e.g., antimicrobial, antioxidant, anti-inflammatory, antiviral, insecticidal and immunomodulatory) and are widely used in health and medicine, cosmetics, perfumes, soaps, plastic resins and foods (as flavorings and preservatives), as well as in aromatherapy as therapeutic agents^2,3^. During distillation, a widely used extraction method for obtaining VOs, plant material is heated with water or steam to vaporize volatile compounds and then condensed. Hydrodistillation (HD) can modify the composition of an oil by polymerizing, isomerizing, or saponifying the more unstable components^4^. Factors affecting the yield of VOs are the type of raw material, size, pH value, raw material quality, equipment used, accuracy, distillation process, cultivation location and distillation time^5^. Drying generally concentrates volatiles and can increase yield^6^. For instance, Hazrati et al.^7^ reported that drying a plant in the shade maximizes VO yield and alters its composition, and that fresh and dried samples have different dominant components. Factors such as harvest conditions, geographic regions, pressure, temperature, and agitation, as well as plant species and parts, also impact yield and VO profile. Additionally, the duration of HD can affect the composition of the VO^4^. Moreover, shortening the distillation time can improve quality. Conversely, excessively long distillation can lead to the degradation of heat-sensitive compounds and deterioration of VO quality^6,8,9^.

The genus Salvia L., belonging to the Lamiaceae family, which generally has plants containing VOs, is used in culinary, medical and cosmetic fields and is found in Europe around the Mediterranean, Southeast Asia and Central and South America^10–12^. *S. officinalis* L., a species of the Salvia genus, is a perennial, evergreen shrub native to the Mediterranean region. Sage has been used in folk remedies to reduce sweating, soothe sore throats, improve menstrual cycles, relieve hot flashes, combat infections (gastroenteritis, intestinal worms), and aid digestion and cognitive function.^9^ Sage products include dried leaves (tea, spice), tinctures, extracts, and VO. *S. officinalis* VO exhibits antimicrobial and antioxidant activity, and its aroma can improve mood and cognitive functions^13,14^. *S. fruticosa* is a perennial shrub native to the Eastern Mediterranean^12^. *S. fruticosa* Mill. (Synonym: S. triloba), known as “Greek sage,” “Anatolian sage,” and “Dalmatian sage,” is a culinary herb used for its medicinal benefits^15^. It is valued in traditional medicine; leaf infusions are used for headaches, rheumatism, heart and stomach ailments, colds, coughs, and indigestion^12^. In Greece and Türkiye, its dried leaves are brewed as “faskomelo” tea, known for its medicinal properties^16^. Due to strong evidence of its beneficial effects, *S. fruticosa* is recognized in the European and British Pharmacopoeias^17^. Products include herbal teas, VOs, and extracts rich in phenolic substances. *S. fruticosa* contains bioactive compounds (abietane and labdane diterpenoids, flavonoids, etc.) responsible for its sedative, carminative, antiseptic, and healing effects^12^. Studies confirm that its VOs and extracts have anti-inflammatory, antioxidant and antimicrobial effects^14^. The VOs of both species are rich in monoterpenes and oxygenated monoterpenes. *S. officinalis* VO typically contains high levels of α- and β-thujone, camphor, 1,8-cineole, and borneol; the main chemotypes are generally “camphor > α-thujone > 1,8-cineole” or “α-thujone-rich.” *S. fruticosa* VO is characteristically rich in 1,8-cineole and camphor. The VOs of the two species share common components (e.g., camphor, α-pinene, β-pinene), albeit in different proportions^9^. The VOs of both plants have antimicrobial, antioxidant, and anti-inflammatory properties, making them beneficial for health and nutrition^13,14^.

Although the effects of post-harvest processing and HD conditions on Salvia VOs have been widely investigated, existing reports often address these factors in isolation^15–17^. For *S. officinalis*, HD time has been evaluated mainly on dried/commercial material and frequently in the context of bioactivity rather than a joint assessment with fresh material across a consistent time series^16^. Likewise, several studies have examined the impact of drying on sage VOs, yet without providing a time-resolved compositional trajectory under a standardized HD regime.^17,18^ For *S. fruticosa*, many reports use a fixed HD time (commonly ~ 3 h) rather than mapping systematic time-dependent compositional shifts^12,19^. Moreover, a number of studies explore alternative extraction/distillation approaches (e.g., microwave-assisted distillation or micro-steam distillation–SPME) that primarily aim at improved processing efficiency, including reduced distillation time^20,21^.

Taken together, a standardized, time-resolved comparison of fresh versus dried material across a consistent HD time series remains limited for these two Salvia species. Accordingly, the present work provides an integrated, standardized comparison (1–5 h) of VO content and major constituent profiles for fresh versus dried leaves of *S. officinalis* and *S. fruticosa* under identical HD and analytical conditions. To this end, fresh and dried leaves were subjected to five HD durations (1, 2, 3, 4, and 5 h) to characterize time-dependent changes in VO content and composition within a practically feasible laboratory distillation window under the applied conditions.

## Materials and methods

### Plant material, location

The plant material was 1-year-old *S. officinalis* L. (common sage) and *S. fruticosa* Mill. (Anatolian sage). For each species, whole plants were cut at approximately 8–10 cm above ground level, and the leaves used for analysis were obtained from the aerial parts of the plants. Leaves were not selectively excluded by plant level (lower, middle, or upper canopy); instead, leaves from the harvested aerial parts were pooled to provide a representative sample of the plant material collected at harvest. Harvesting was performed at the onset of flowering (when the first flowers were observed in the field); however, flowering shoots/inflorescences were not collected, and only leaves were used, in order to focus the study on leaf VO characteristics. *S. officinalis* and *S. fruticosa* samples were obtained from Medicinal and Aromatic Plants Research and Application Field of Afyonkarahisar Health Sciences University (38°46’ N, 30°27’ E, 1009 m) on July 10 and 23, 2023, respectively. This area is located in Turkey’s Inner Aegean region. Inner Aegean Region; it is known as the transitional climate zones, which is affected by three main climatic conditions: Mediterranean (South), Central Anatolia (East) and Aegean (West) regions. The climate of the experimental location is harsh with moderate rainfall. Most precipitation occurs in winter and spring. Summers are hot and dry and winters are cold and snowy^22^. Table 1 shows some of the local meteorological data during the plant growing season. Also, the physicochemical properties of the soil are given in Table 2.

**Table 1:** Local meteorological data in which the plants were grown.

	Jan.	Feb.	Mar.	Apr.	May	Jun.	Jul.
Min. Temperature (°C)	– 0.9	-3.3	2.6	5	9.1	13	15.2
Max Temperature (°C)	9.1	7.2	12.7	15	19.8	24.3	30.8
Mean Temperature (°C)	3.4	1.5	7.3	9.7	13.7	18.3	23.2
Rain (mm)	20.1	11.5	60.6	65.9	204.3	80.2	10.5
Relative humidity (%)	75.3	63.6	67.3	63.1	73.1	67.5	45.4
Insolation (h)	145	145.3	139.8	145	162.3	248	368.5

**Table 2:** The values of some physicochemical properties of the experimental soil.

Properties	Values
Organic matter (%)	1.18
Salt (%)	0.062
CaCO₃ (%)	6.49
Field capacity (%)	69.54
EC (mS cm^-1^)	0.099
pH	7.67
Elements	
P (kg ha^-1^)	126.0
K (kg ha^-1^)	476.3
Ca (mg kg^-1^)	5159
Mg (mg kg^-1^)	864
Fe (mg kg^-1^)	5.362
Cu (mg kg^-1^)	0.425
Zn (mg kg^-1^)	0.521
Mn (mg kg^-1^)	1.965
Soil class: Clay-Loam.	

### Volatile oil hydrodistillation

To investigate time-dependent changes in VO content during hydrodistillation (HD), five predefined distillation times were selected (1, 2, 3, 4, and 5 h). The 1–5 h window was chosen to capture early distillate stages (1–2 h), the commonly used mid-range duration (≈ 3 h), and extended times (4–5 h) to evaluate diminishing returns in content and the emergence of later-eluting, less volatile constituents within a practically feasible laboratory HD period. Fresh leaves of *S. officinalis* and *S. fruticosa* were hydrodistilled as whole leaves immediately after cutting. For dried samples, leaves were separated from stems, spread as a single layer, and dried at 37 °C for 72 h in a drying/heating chamber with natural convection (Binder ED 720). The dried leaves were then manually fragmented to obtain a particle size of approximately 2–5 mm prior to hydrodistillation. For each condition (species × leaf state × HD time), 50 g of plant material was hydrodistilled with distilled water (1:10, w/v) using a Clevenger-type apparatus, and VO content was determined from two independent hydrodistillations (*n* = 2). VO content was determined volumetrically and expressed as mL 100 g⁻¹ (fresh or dried weight, as applicable). After decantation, the oils were dried over anhydrous sodium sulfate and stored in amber vials at 4 °C until analysis.

### Identification of volatile oil compounds

A GC/FID-MS system (Shimadzu GC-MS QP2010 SE) was used to characterize the chemical composition of the VOs. Sample preparation, column specifications, and the GC/FID-MS operating and injection conditions are summarized in Table 3. Retention indices (RI) were calculated using a C_7_–C_30_ *n*-alkane series (Merck/Supelco) analyzed under the same conditions as the VO samples. Compound identification was achieved by comparing RI values and mass spectra with the NIST and Wiley libraries, published mass spectral data^23^, and an in-house database. Relative abundance (% area) was calculated by area normalization (peak area of each compound divided by the sum of peak areas of all identified compounds), and no individual response factors were applied. As compositional profiling was obtained from a single chromatographic run per condition, compositional comparisons are presented descriptively and no inferential statistics were applied to the compositional data.

**Table 3:** Column specifications and GC/FID-MS analysis conditions.

GC/FID-MS analysis conditions	
Stationary phase	Teknokroma, TRB-WAX (Shimadzu GC-MS QP2010 SE)
Stationary phase specifications	60 m × 0.320 mm inner diameter and 0.25 μm film thickness
Carrier gas / Flow	Helium (99.999%) / 2.06 mL min⁻¹
Sample preparation	1 ml: 1:50 / VO diluted in n-Hexane
Injection volume	1 µl (split mode, 40:1)
1st stage temp. planning & gradient	70 °C after injecting with 5 min hold-up time, after raised to 160 °C with a 3 °C min^− 1^ heating ramp and 10 min hold-up time
2nd stage temp. planning & gradient	Increased to 250 °C with 6 °C min^− 1^ heating ramp and 20 min hold time
Detector temp.	250 °C
Injector temp.	250 °C
Ion source temp.	250 °C
MS scan range (m z^− 1^)	35–550 atomic mass units (AMU)
Electron impact ionization	70 eV

### Statistical and chemometric analysis

VO content values are reported as mean ± SD (*n* = 2 independent hydrodistillations). Content relative to maximum content (%) was calculated, for each species and material state (FS or DS), by dividing the VO content at each HD time by the maximum VO content observed within that material state and multiplying by 100. The rate of increase (DS/FS) was calculated at each HD time as the ratio of DS to FS VO content (dimensionless). Given the limited replication and the lack of independent replicate extractions/GC profiles for compositional data, no inferential statistics (p-values) were applied; comparisons are interpreted descriptively under the applied conditions.

Principal component analysis (PCA) was employed as an exploratory chemometric approach to characterize and compare the volatile-oil (VO) profiles obtained from fresh and dried leaves of *S. officinalis* and *S. fruticosa* after HD for 1, 2, 3, 4, and 5 h. For each species, PCA was conducted separately using the relative abundances of the selected major constituents (expressed as % of total peak area from GC–FID/MS) for the ten conditions (fresh vs. dried × five distillation times). The analysis was performed on the correlation matrix of the selected variables, and the first two principal components were used for interpretation. PCA biplots (PC1 vs. PC2) were generated to visualize similarities among samples and the contribution of major compounds, summarizing the combined effects of leaf condition and HD time on the volatile profiles. Pearson’s correlation coefficients (r) among major constituents were also calculated. All multivariate analyses were performed using JMP 11 (SAS Institute Inc., 2014). Given the lack of independent replicate extractions/analytical measurements for compositional profiling, no inferential statistics were applied to the compositional (% peak area) data; therefore, PCA outputs are interpreted descriptively.

## Results and discussions

### Volatile oil content (%)

Table 4 presents the VO content (%) of fresh and dried *S. officinalis* samples subjected to HD for different durations (1–5 h). In fresh samples, VO content increased steadily with prolonged HD, ranging from 0.37% at 1 h to 0.64% at 5 h. Taking the 5-hour VO content as 100%, the relative VO content values obtained after 1, 2, 3, and 4 h corresponded to 57.8%, 75.0%, 78.1%, and 93.8%, respectively. Similarly, in dried *S. officinalis* samples, the VO content rose from 1.48% at 1 h to 2.00% at 5 h, with relative VO content values of 74.0%, 82.0%, 90.0%, and 90.0% for the respective time intervals. Drying the plant material led to a 66.6% reduction in fresh weight, implying a threefold concentration due to moisture loss. Despite this reduction, the VO content values in dried samples were consistently higher than those in fresh samples at each time point. Specifically, at 1, 2, and 3 h, the VO content values of dried samples were approximately 4.00, 3.42, and 3.60 times higher, respectively, than those of fresh material. These ratios highlight the enhanced extraction efficiency associated with the dried matrix. Considering the moisture loss during drying, VO content values were higher in dried material than in fresh material, especially within the first three hours of HD. Notably, the VO content in dried samples plateaued after the third hour, with minimal or no further increase observed in the 4- and 5-hour treatments. Therefore, under the present experimental conditions, 3 h can be considered a practically sufficient duration for dried leaves, as ~ 90% of the 5-h content was recovered by 3 h and further increases at 4–5 h were minimal; however, longer times may be selected when the aim is to maximize total recovery. In fresh leaves, VO content continued to increase up to 5 h; thus, the results are interpreted as time-dependent trends rather than a single “optimal” duration.

**Table 4:** Volatile oil content (%) of fresh and dried samples of Salvia species affected by various hydrodistillation durations. (Values are mean ± SD / *n* = 2 independent hydrodistillations).

Samples	Hydrodistillation time				
	1 h	2 h	3 h	4 h	5 h
	S. officinalis				
VO of Fresh samples (FS / %)	0.37 ± 0.02	0.48 ± 0.01	0.50 ± 0.01	0.60 ± 0.00	0.64 ± 0.01
Content relative to max. content (%)	57.8	75.0	78.1	93.8	100.0
VO of Dried samples (DS / %)	1.48 ± 0.03	1.64 ± 0.02	1.80 ± 0.00	1.80 ± 0.02	2.00 ± 0.02
Content relative to max. content (%)	74	82	90	90	100
Rate of increase (DS FS^− 1^)	4.00	3.42	3.60	3.00	3.13
	*S. fruticosa*				
VO of Fresh samples (FS / %)	0.42 ± 0.01	0.46 ± 0.02	0.46 ± 0.02	0.50 ± 0.02	0.50 ± 0.01
Content relative to max. content (%)	84	92	92	100	100
VO of Dried samples (DS / %)	1.20 ± 0.02	1.40 ± 0.03	1.52 ± 0.01	1.52 ± 0.04	1.52 ± 0.03
Content relative to max. content (%)	78.9	92.1	100	100	100
Rate of increase (DS FS^− 1^)	2.86	3.04	3.30	3.04	3.04

SD values may round to 0.00 when reported to two decimals.

*S. fruticosa* exhibited a slight increase in VO content in fresh samples with extended HD durations, ranging from 0.42% at 1 h to 0.50% at 5 h (Table 4). However, the VO content remained constant at 0.46% during both the 2- and 3-hour HD. Although a slight increase of 0.04% was observed at both the 4- and 5-hour time points compared to the 2- and 3-hour durations, the VO content plateaued at 0.50%. Considering the VO content at 4 and 5 h as 100%, the relative VO content values obtained after 1, 2, and 3 h of HD corresponded to 84%, 92%, and 92%, respectively. Similarly, in the dried *S. fruticosa* material, the VO content increased during the initial three hours of treatment, rising from 1.20% at 1 h to 1.40% at 2 h and 1.52% at 3 h. However, no further increase was observed at 4 and 5 h, with VO content remaining constant at 1.52%. Accordingly, the VO content at 1 and 2 h corresponded to 78.9% and 92.1% of the maximum content obtained from the 3-, 4-, and 5-hour HD, respectively. Drying resulted in a 74.6% reduction in the weight of *S. fruticosa* leaves, corresponding to an approximate 3.9-fold concentration due to moisture loss. Despite the weight loss, VO content values in dried samples were 2.86 to 3.30 times higher than those in fresh samples (at the corresponding HD times). However, after adjusting for the 3.9-fold moisture loss, the relative VO content advantage of dried samples was reduced. Therefore, when considering the VO content relative to the original fresh weight, fresh *S. fruticosa* leaves may offer a more favorable outcome.

The gradual increase in VO content with prolonged HD time may be attributed to the slower evaporation and subsequent condensation of higher-boiling-point compounds, which are eventually incorporated into the VO. HD time plays a crucial role in determining both the content and chemical profile of VOs. Low-boiling-point monoterpenes are typically extracted rapidly, whereas higher molecular weight compounds such as sesquiterpenes are gradually liberated over extended durations. However, this time-dependent increase is more pronounced in *S. officinalis*, whereas *S. fruticosa* reaches its maximum VO content within a shorter HD period, indicating species-specific extraction dynamics. For instance, a study on rosemary reported that 37% of the total VO was obtained within the initial 10 min, 61.5% by 20 min, 75% by 30 min, 87% by 60 min, and the full yield was obtained within 120 minutes^24^. Additionally, the gradual breakdown of oil glands within the plant tissue and the kinetics of the extraction process lead to the release of different components at varying intervals. However, extended HD or steam distillation may alter the compositional profile, increasing the risk of thermal degradation, oxidation, or hydrolysis of sensitive compounds. Therefore, while VO yield typically increases over time and eventually approaches a plateau, the final compositional profile can vary depending on the process^7,25^.

According to the European Medicines Agency^26^, the dried leaves of *S. officinalis* must contain at least 12 mL kg⁻¹ of VO in whole form and 10 mL kg⁻¹ in cut form. For *S. fruticosa*, which is also used medicinally, the VO content is expected to be 18 mL kg⁻¹ in whole dried leaves and 12 mL kg⁻¹ in cut dried leaves^27^. The observed differences in *S. fruticosa* can be attributed to the substantial moisture loss during drying, which increases the VO concentration on a dry weight basis. However, the actual content may be lower than that of fresh material due to the potential degradation or loss of certain volatile compounds during the drying process. Fresh medicinal and aromatic plants typically contain 75–80% moisture. If they are not processed fresh, this level must be reduced to below 15% to prevent biochemical changes and microbial spoilage. Drying is widely used for this purpose. However, the VO content and composition of any medicinal and aromatic plant can vary significantly depending on the specific drying method and processing parameters^28^. While drying leads to a relative increase in VO concentration due to water loss, the applied temperature, oxidative conditions, and enzymatic activity can cause degradation or transformation of especially sensitive monoterpenes. Different drying methods and durations may thus alter the relative composition of these constituents. Furthermore, the duration of HD or steam distillation plays a critical role in determining the extent of cell disruption and the kinetics of compound extraction. Typically, this process facilitates the rapid release of highly volatile compounds during the initial stages, followed by the slower extraction of heavier or less volatile compounds as the distillation progresses^7,25^.

### Volatile oil component

Table 5 presents the VO composition (%) of both fresh and dried *S. officinalis* samples subjected to varying HD durations. GC/FID-MS analysis identified 41 compounds in the fresh samples and 32 in the dried ones across all HD times. α-Thujone was the major constituent in the VOs of both fresh and dried samples. Notably, the α-thujone content in dried samples decreased as the HD time increased. The highest α-thujone levels were observed at 1-hour (F: 25.01%; D: 22.62%), decreasing steadily to their lowest at 5 h (F: 17.1%; D: 18%). Other major components identified in both fresh and dried samples included camphor (14.09–19.92%), 1,8-cineole (9.55–14.5%), borneol (8.52–12.13%), β-thujone (4.4–5.72%), camphene (3.42–4.9%), α-pinene (2.89–6.23%), bornyl acetate (2.69–3.87%), viridiflorol (2.1–8.23%), caryophyllene (1.66–4.24%), α-humulene (1.39–4.21%), β-pinene (1.36–1.79%), and limonene (1.49–1.97%). In both fresh and dried samples, 1,8-cineole levels decreased with prolonged HD durations. In fresh samples, 1,8-cineole ranged from 14.5% at 1 h to 9.55% at 5 h; in dried samples, it decreased from 13.24% to 10.71% over the same period. Viridiflorol, an oxygenated sesquiterpene, generally increased with longer HD durations, peaking at 4 h in both sample types. The highest concentrations were found in the 4-hour procedure (F: 8.23%; D: 5.98%). Although most minor components remained relatively stable, some—such as linalool and linalyl acetate—showed notable decreases over time. Linalool (0.35–1.92%) was identified in all samples. In fresh samples, linalool levels were highest during the initial two HD times (1 h: 1.85%; 2 h: 1.92%) before sharply decreasing to concentrations ranging from 0.35% to 0.64% in subsequent treatments. The presence of other minor compounds was also time-dependent. Linalyl acetate was detected in fresh samples during the initial three HD times, with its concentration decreasing over time (1 h: 1.64%; 2 h: 0.7%; 3 h: 0.08%). Lavandulyl acetate (0.09–0.46%), sabinyl acetate (0.11–0.15%), β-farnesene (0.18–0.73%), and γ-eudesmol (0.07–0.2%) were detected during the initial four HD periods of the fresh samples. Geranyl acetate was present in the initial three HD times of fresh samples (0.14–0.44%) and only at the 3-hour mark in dried samples (0.12%). Neryl acetate (0.15–0.28%) and geraniol (0.24–0.27%) were found only during the initial two HD times of the fresh samples, while nerol was detected exclusively at the 1-hour mark (0.1%). In contrast, trans-β-farnesene (0.1–0.16%), myrtenyl acetate (0.09–0.33%), and epimanool (0.81–5.68%) were absent in the 1-hour HD of the fresh samples (Table 5). The observed variations in *S. officinalis* VO composition with HD time and drying status underscore the dynamic nature of VO extraction, highlighting that distillation duration should be selected according to the intended compositional profile and practical processing constraints. Given that major constituents such as α-thujone, camphor, and 1,8-cineole shift across HD times, the results indicate that extraction time can reshape the recovered VO profile through time-dependent fractionation of volatiles. Extended HD may enrich later-eluting, less volatile constituents while reducing the relative abundance of early-recovered monoterpenes, potentially reflecting differential extraction kinetics and partial thermal/oxidative transformations. Accordingly, within the investigated 1–5 h window, HD duration is best presented as a process-time variable that influences the balance between incremental content gains and compositional shifts under the applied conditions. Moreover, the differences between fresh and dried samples indicate that moisture content and post-harvest processing may modify the plant matrix (e.g., tissue permeability and volatilization losses) during hydrodistillation, thereby affecting the recovered volatile profile.

**Table 5:** Volatile oil compounds of fresh and dried samples of *S. officinalis* affected by various hydrodistillation durations (% of total peak area; GC-FID/MS).

RT^1^	RI^2^	RI^3^	Compounds	G.C.^4^	Hydrodistillation duration of fresh samples (F)					Hydrodistillation duration of dried samples (D)				
					1 h	2 h	3 h	4 h	5 h	1 h	2 h	3 h	4 h	5 h
8.556	1020	1019^43^	Tricyclene	MH^5^	0.09	0.09	0.1	0.11	0.11	0.14	0.14	0.12	0.13	0.15
8.819	1032	1031^43^	α-Pinene	MH	2.89	3.8	3.56	3.68	4.72	5.58	5.57	5.2	5.78	6.23
9.849	1079	1076^43^	Camphene	MH	3.42	3.52	3.45	3.73	3.67	4.88	4.78	4.38	4.48	4.9
10.936	1120	1120^43^	β-Pinene	MH	1.63	1.36	1.6	1.71	1.52	1.75	1.75	1.6	1.63	1.79
11.257	1131	1130^43^	Sabinene	MH	0.15	0.12	0.19	0.2	0.21	0.14	0.15	0.14	0.14	0.15
12.413	1168	1167^43^	β-Myrcene	MH	0.74	0.66	0.76	0.79	0.72	0.8	0.81	0.76	0.78	0.84
13.116	1190	1190^43^	α-Terpinene	MH	0.11	0.07	0.12	0.11	0.1	0.15	0.15	0.16	0.15	0.16
13.774	1209	1209^43^	Limonene	MH	1.55	1.49	1.6	1.65	1.73	1.77	1.86	1.82	1.87	1.97
14.152	1219	1219^43^	1,8-Cineole	OM^6^	14.5	12.23	10.77	9.55	9.82	13.24	12.21	11.39	10.71	10.86
15.468	1254	1254^43^	γ-Terpinene	MH	0.21	0.15	0.24	0.24	0.21	0.28	0.29	0.3	0.29	0.31
16.475	1281	1281^43^	p-Cymene	MH	0.26	0.19	0.23	0.24	0.18	0.22	0.23	0.21	0.2	0.21
16.921	1292	1292^43^	α-Terpinolen	MH	0.27	0.27	0.3	0.31	0.37	0.34	0.35	0.37	0.38	0.4
22.735	1435	1435^43^	α-Thujone	OM	25.01	20.72	21.83	20.83	17.1	22.62	21.32	19.94	18.16	18
23.473	1453	1454^43^	β-Thujone	OM	5.11	5.22	4.41	4.4	4.73	5.72	5.3	5.3	4.91	5.04
24.12	1469	1469^44^	*cis*-Sabinene hydrate	OM	0.23	0.27	0.22	0.21	0.25	0.18	0.19	0.15	0.18	0.17
26.574	1530	1530^43^	Camphor	OM	15.57	16.12	14.97	14.09	15.42	19.92	17.81	17.92	16.42	16.96
27.358	1550	1550^43^	Linalool	OM	1.85	1.92	0.64	0.54	0.35	0.42	0.4	0.43	0.41	0.38
27.496	1553	1554^44^	*trans*-Sabinene hydrate	OM	0.16	0.2	0.17	0.15	0.2	0.16	0.17	0.15	0.16	0.15
27.845	1562	1563^44^	Linalyl acetate	E^7^	1.64	0.7	0.08							
28.949	1590	1590^43^	Bornyl acetate	E	2.87	3.59	2.69	3.08	3.66	2.94	3.6	3.84	3.87	3.77
29.67	1608	1608^43^	Caryophyllene	SH^8^	2.54	2.62	4.24	3.58	2.92	1.66	1.93	1.97	2.38	2.39
29.802	1612	1611^44^	Lavandulyl acetate	E	0.41	0.46	0.09	0.09						
31.581	1659	1659^43^	Sabinyl acetate	E	0.15	0.11	0.12	0.13						
31.862	1666	1664^45^	Thujol	OM	0.19	0.28	0.21	0.2	0.27	0.32	0.27	0.3	0.28	0.29
32.045	1671	1671^4^	β-Famesene	SH	0.18	0.73	0.42	0.45						
32.319	1678	1670^44^	*trans*-β-Farnesene	SH		0.13	0.1	0.1	0.14	0.14	0.13	0.16	0.15	0.13
32.434	1681	1681^43^	α-Humulene	SH	2.27	1.6	4.21	3.86	3.39	1.39	1.66	1.84	2.36	2.39
33.023	1697	1698^46^	Myrtenyl acetate	E		0.09	0.33	0.11	0.14	0.09	0.12	0.13	0.12	0.12
33.241	1702	1703^43^	α-Terpineol	OM	0.71	0.79		0.24	0.33	0.34	0.35	0.43	0.36	0.35
33.452	1708	1709^43^	Borneol	OM	9.75	11.96	8.52	8.72	10.6	10.75	12.13	11.58	11.51	10.24
33.898	1720	1716^44^	Germacrene D	SH		0.15	0.09	0.13						
34.305	1731	1731^44^	Neryl acetate	E	0.15	0.28								
35.409	1760	1760^44^	Geranyl acetate	E	0.27	0.44	0.14					0.12		
36.817	1797	1798^44^	Myrtenol	OM	0.15	0.34	0.24	0.2	0.38	0.34	0.37	0.38	0.38	0.38
37.017	1802	1803^44^	Nerol	OM	0.1									
38.945	1846	1847^44^	Geraniol	OM	0.27	0.24								
44.719	1995	1995^43^	Caryophyllene oxide	OS^9^	0.54	0.57	0.88	1	0.79	0.31	0.53	0.59	0.71	0.66
46.43	2053	2053^43^	Humulene epoxide II	OS	0.39	0.42	0.81	0.83	0.8	0.31	0.48	0.56	0.72	0.67
47.54	2093	2093^43^	Viridiflorol	OS	2.73	2.99	7.14	8.23	7.84	2.1	3.22	4.38	5.98	5.81
49.623	2179	2179^47^	γ-Eudesmol	OS	0.09	0.2	0.1	0.07						
58.892	2691	2691^43^	Epimanool	OD^10^		1.09	3.14	5.02	5.68	0.81	1.12	2.01	3.17	2.84
Grouped compounds (%)														
Monoterpene hydrocarbons					11.32	11.72	12.15	12.77	13.54	16.05	16.08	15.06	15.83	17.11
Oxygenated monoterpenes					73.6	70.29	61.98	59.13	59.45	74.01	70.52	67.97	63.48	62.82
Sesquiterpene hydrocarbons					4.99	5.23	9.06	8.12	6.45	3.19	3.72	3.97	4.89	4.91
Oxygenated sesquiterpene					3.75	4.18	8.93	10.13	9.43	2.72	4.23	5.53	7.41	7.14
Oxygenated diterpenoids						1.09	3.14	5.02	5.68	0.81	1.12	2.01	3.17	2.84
Esters					5.49	5.67	3.45	3.41	3.8	3.03	3.72	4.09	3.99	3.89
Total (%)					99.15	98.18	98.71	98.58	98.35	99.81	99.39	98.63	98.77	98.71

^1^Retention time; ^2^Retention indices calculated against n-alkanes (C7-C30) on HP-Innowax column; ^3^RI; ^4^Grouped compounds; ^5^Monoterpene hydrocarbons; ^6^Oxygenated monoterpenes; ^7^Ester; ^8^Sesquiterpene hydrocarbons; ^9^Oxygenated sesquiterpene; ^10^Oxygenated diterpenoids.

The VO components of *S. officinalis* were categorized into six chemical groups: monoterpene hydrocarbons (ranging from 11.32% to 17.11%), oxygenated monoterpenes (59.13–74.01%), sesquiterpene hydrocarbons (3.19–9.06%), oxygenated sesquiterpenes (2.72–10.13%), oxygenated diterpenoids (0.81–5.68%), and esters (3.03–5.67%). Oxygenated monoterpenes were the predominant group among them, exhibiting a decreasing trend as HD time increased in both fresh and dried samples. Monoterpene hydrocarbons exhibited a similar pattern in fresh samples, showing an increase with extended HD time, while no marked changes were observed in the dried samples under the applied conditions. The concentration of sesquiterpene hydrocarbons differed between the fresh and dried samples. In fresh samples, the highest concentrations were observed at 3- and 4-hour HD durations. Conversely, the concentration of this group in dried samples steadily increased with longer HD durations. Oxygenated sesquiterpenes increased until the 4-hour HD in both fresh and dried samples but decreased at the 5-hour point. Oxygenated diterpenoids were absent in the 1-hour HD of fresh samples; however, their concentrations increased with prolonged HD times in both fresh and dried samples. Finally, within the esters group, the highest concentrations were recorded in the first two HD of fresh samples (5.49% and 5.67%), whereas the lowest concentration was observed in the initial HD of dried samples (3.03%) (Table 5). The observed decline in oxygenated monoterpenes and concurrent increase in oxygenated sesquiterpenes and diterpenoids over extended HD times may be attributed to differences in volatility and molecular weight among these compound classes. Extended HD time may also promote degradation or transformation of more labile monoterpenes, thus contributing to the relative increase in heavier compound groups. Therefore, selection of HD time should be tailored based on the desired chemical profile, as shorter durations favor monoterpene-rich oils, while longer durations enhance sesquiterpene and diterpenoid content.

Table 6 summarizes the composition (%) of VOs obtained from fresh and dried *S. fruticosa* samples subjected to different HD durations. GC/FID-MS analysis revealed a total of 49 compounds in the VOs from fresh samples and 48 compounds in those from dried samples across five HD durations. α-Thujone was identified as the dominant compound in the VOs of both fresh and dried samples. The maximum α-thujone concentration was recorded at the 1-hour HD point in both fresh (16.66%) and dried (17.19%) samples. Although a downward trend was observed in subsequent treatments, the decrease was modest under the applied conditions. Other notable components in both sample types included 1,8-cineole (8.36–12.45%), caryophyllene (8.54–12.78%), camphor (9.44–13.28%), α-pinene (4.62–9.28%), α-humulene (5.67–8.75%), β-thujone (3.94–5.13%), camphene (2.64–5.06%), epimanool (2.11–8.49%), β-myrcene (1.77–2.72%), β-pinene (1.87–2.72%), aromadendrene (2.07–3.24%), borneol (1.51–1.86%), viridiflorol (1.37–3.59%), limonene (1.08–1.58%), and α-terpineol (0.82–1.38%). The highest level of 1,8-cineole was detected in both fresh and dried samples during the 1-hour HD, with concentrations of 11.81% and 12.45%, respectively. While a gradual decrease was noted in the subsequent HD periods, the decline in 1,8-cineole levels was not substantial. The lowest caryophyllene concentrations were recorded at the 1-hour HD (F: 10.8%; D: 8.54%). While fresh samples exhibited moderate fluctuations over time, dried samples showed a notable increase, peaking at 12.25% after 5 h. Camphor content exhibited distinct trends in fresh and dried samples; it remained relatively stable in fresh samples, whereas a clear decreasing trend was observed in dried samples as the HD time increased. Generally, α-pinene concentrations were higher in dried samples (ranging from 6.62% to 9.28%) compared to fresh samples (4.62% to 7.45%), with peak values occurring at the 1-hour HD in both sample types. With increasing HD time, levels of camphene, β-myrcene, and β-pinene showed a decreasing trend, while epimanool content increased; nevertheless, these changes were irregular and did not exhibit a consistent pattern. The compounds ι-gurjunene, alloaromadendrene, γ-gurjunene, and epiglobulol were not detected in either sample group during the 1-hour HD process. Germacrene D was detected exclusively at the 4-hour HD time in both fresh and dried samples. In summary, the VO composition of *S. fruticosa* varied with HD time and drying state, indicating that distillation-time selection can influence VO content and the relative abundance of key constituents under the applied conditions.

**Table 6:** Volatile oil compounds of fresh and dried samples of *S. fruticosa* affected by various hydrodistillation durations (% of total peak area; GC-FID/MS).

RT^1^	RI^2^	RI^3^	Compounds	G.C.^4^	Hydrodistillation duration of fresh samples (F)					Hydrodistillation duration of dried samples (D)				
					1 h	2 h	3 h	4 h	5 h	1 h	2 h	3 h	4 h	5 h
8.458	1015	1019^43^	Tricyclene	MH^5^	0.26	0.1	0.1	0.08	0.08	0.18	0.15	0.15	0.16	0.12
8.722	1028	1031^43^	α-Pinene	MH	7.45	5.55	5.46	4.62	4.66	9.28	7.79	7.74	8.31	6.62
9.74	1074	1076^43^	Camphene	MH	4.41	3.23	3.16	2.64	2.83	5.06	4.13	4.18	4.44	3.56
10.822	1117	1120^43^	β-Pinene	MH	2.59	2.15	2.14	1.88	1.87	2.72	2.33	2.44	2.49	2.14
11.136	1127	1130^43^	Sabinene	MH	0.38	0.35	0.39	0.4	0.26	0.29	0.28	0.32	0.3	0.29
12.286	1164	1167^43^	β-Myrcene	MH	2.62	2.23	2.21	1.95	1.77	2.72	2.35	2.38	2.42	2.18
12.985	1186	1190^43^	α-Terpinene	MH	0.34	0.3	0.33	0.32	0.34	0.46	0.41	0.44	0.43	0.43
13.637	1206	1209^43^	Limonene	MH	1.36	1.18	1.14	1.08	1.21	1.58	1.38	1.33	1.42	1.27
14.02	1216	1219^43^	1,8-Cineole	OM^6^	11.81	9.51	8.77	8.36	8.78	12.45	10.52	10.66	10.43	9.52
14.781	1236	1240^44^	*cis*-Ocimene	MH	0.1	0.1	0.11	0.1	0.09	0.1	0.11	0.12	0.1	0.11
15.325	1250	1254^43^	γ-Terpinene	MH	0.66	0.6	0.64	0.63	0.66	0.82	0.76	0.81	0.8	0.78
16.326	1277	1281^43^	p-Cymene	MH	0.28	0.27	0.26	0.26	0.4	0.43	0.37	0.35	0.38	0.34
16.772	1288	1292^43^	α-Terpinolene	MH	0.33	0.29	0.29	0.29	0.29	0.36	0.33	0.33	0.33	0.32
22.575	1431	1435^43^	α-Thujone	OM	16.66	14.51	14.18	14.28	15.08	17.19	15.19	15.4	15.14	13.94
23.324	1450	1454^43^	β-Thujone	OM	4.71	4.18	4.02	3.94	4.78	5.13	4.54	4.54	4.55	4.24
23.982	1466	1469^44^	*cis*-Sabinene hydrate	OM	0.27	0.24	0.21	0.25	0.16	0.23	0.25	0.23	0.25	0.22
24.303	1474	NF*	Isoledene	SH^7^	0.15	0.16	0.17	0.18	0.16	0.15	0.18	0.18	0.18	0.21
26.414	1526	1530^43^	Camphor	OM	10.66	9.94	9.44	9.8	11.57	13.28	12.84	11.37	11.28	10.59
26.826	1536	1546^48^	α-Gurjunene	SH	0.13	0.13	0.14	0.15	0.13	0.12	0.15	0.15	0.15	0.18
27.221	1546	1550^43^	Linalool	OM	0.45	0.45	0.41	0.44	0.5	0.52	0.45	0.43	0.45	0.44
27.358	1550	1554^44^	*trans*-Sabinene hydrate	OM	0.25	0.26	0.25	0.29	0.22	0.25	0.3	0.27	0.28	0.28
28.806	1586	1590^43^	Bornyl acetate	E^8^	0.87	0.79	0.8	0.79	0.61	0.59	0.64	0.75	0.58	0.58
29.321	1599	NF	ι-Gurjunene	SH		0.14	0.15	0.17	0.14		0.15	0.16	0.17	0.18
29.544	1605	1608^43^	Caryophyllene	SH	10.8	11.56	12.2	12.78	11.58	8.54	10.81	11.06	10.78	12.5
29.698	1609	1610^49^	β-Gurjunene	SH	0.54	0.58	0.62	0.65	0.58	0.47	0.61	0.62	0.6	0.73
29.904	1615	1618^43^	Aromadendrene	SH	2.4	2.6	2.74	2.92	2.62	2.07	2.7	2.79	2.91	3.24
30.225	1623	1666^50^	β-Selinene	SH	0.28	0.31	0.33	0.35	0.32	0.25	0.33	0.34	0.32	0.39
31.364	1653	1655^51^	Alloaromadendrene	SH		0.07	0.15	0.15	0.05		0.07	0.07	0.24	0.24
31.713	1662	1664^52^	γ-Gurjunene	SH		0.13	0.11	0.13	0.13		0.13	0.13	0.14	0.12
32.176	1674	1670^44^	trans-β-Farnesene	SH	0.12	0.14	0.12	0.15	0.17	0.15	0.17	0.16	0.16	0.18
32.308	1678	1681^43^	α-Humulene	SH	7.14	8.24	8.6	8.75	7.75	5.67	7.35	7.47	7.83	8.45
32.886	1693	1698^53^	Myrtenyl acetate	E	0.16	0.15	0.16	0.18	0.13	0.1	0.15	0.15	0.14	0.15
33.092	1698	1753^54^	α-Chamigrene	SH	0.43	0.49	0.45	0.52	0.56	0.46	0.56	0.53	0.56	0.59
33.195	1701	1703^43^	α-Terpineol	OM	1.01	1.12	1.17	1.25	1.08	0.82	1.15	1.19	1.11	1.38
33.297	1704	1709^43^	Borneol	OM	1.51	1.86	1.55	1.53	1.85	1.73	1.74	1.66	1.64	1.51
33.755	1716	1716^44^	Germacrene D	SH				0.41					0.13	
34.642	1740	1755^55^	Bicyclogermacrene	SH	0.86	1	1.09	1.13	0.8	0.13	0.21	0.22	0.19	0.21
35.415	1760	1765^55^	δ-Cadinene	SH			0.1	0.14	0.1					0.1
36.668	1793	1798^44^	Myrtenol	OM	0.25	0.31	0.29	0.3	0.33	0.27	0.31	0.29	0.31	0.28
44.593	1991	1995^43^	Caryophyllene oxide	OS^9^	0.55	1.09	0.85	0.74	1.01	0.35	0.64	0.57	0.61	0.63
45.394	2017	2025^53^	Epiglobulol	OS		0.13	0.13	0.19	0.14		0.1	0.1	0.1	0.13
46.309	2049	2053^43^	Humulene epoxide II	OS	0.46	1.08	0.77	0.85	0.84	0.35	0.56	0.53	0.52	0.55
47.191	2080	2075^56^	Globulol	OS	0.27	0.44	0.45	0.5	0.48	0.12	0.36	0.33	0.35	0.42
47.431	2089	2093^43^	Viridiflorol	OS	1.41	3.59	2.91	1.78	1.84	1.49	1.47	1.66	1.37	1.63
47.997	2110	2144^57^	Rosifoliol	OS	0.12	0.19	0.23	0.2	0.21		0.17	0.15	0.16	0.19
48.426	2129	2133^55^	Spathulenol	OS	0.33	0.59	0.54	0.57	0.63	0.2	0.49	0.44	0.48	0.54
49.68	2182	NF	ent-Pimara-8,15-diene	DH^10^	0.24	0.26	0.27	0.39	0.27			0.19	0.2	0.29
50.864	2237	2230^49^	τ-Muurolol	OS			0.07	0.25	0.08					
58.795	2686	2691^43^	Epimanool	OD^11^	3.15	5.76	7.62	8.49	8.25	2.11	2.68	2.88	3.05	5.38
Grouped compounds (%)														
Monoterpene hydrocarbons					20.78	16.35	16.23	14.25	14.46	24	20.39	20.59	21.58	18.16
Oxygenated monoterpenes					47.58	42.38	40.29	40.44	44.35	51.87	47.29	46.04	45.44	42.4
Sesquiterpene hydrocarbons					22.85	25.55	26.97	28.58	25.09	18.01	23.42	23.88	24.36	27.32
Oxygenated sesquiterpene					3.14	7.11	5.95	5.08	5.23	2.51	3.79	3.78	3.59	4.09
Diterpenoid hydrocarbons					0.24	0.26	0.27	0.39	0.27			0.19	0.2	0.29
Oxygenated diterpenoids					3.15	5.76	7.62	8.49	8.25	2.11	2.68	2.88	3.05	5.38
Esters					1.03	0.94	0.96	0.97	0.74	0.69	0.79	0.9	0.72	0.73
Total (%)					98.77	98.35	98.29	98.2	98.39	99.19	98.36	98.26	98.94	98.37

^1^Retention time; ^2^Retention indices calculated against n-alkanes (C7-C30) on HP-Innowax column; ^3^RI; ^4^Grouped compounds; ^5^Monoterpene hydrocarbons; ^6^Oxygenated monoterpenes; ^7^Sesquiterpene hydrocarbons; ^8^ Ester; ^9^Oxygenated sesquiterpene; ^10^Diterpenoid hydrocarbons; ^11^Oxygenated diterpenoids; *Not found.

The chemical classes identified in the VO of *S. fruticosa* comprised monoterpene hydrocarbons (14.25-24%), oxygenated monoterpenes (40.29–51.87%), sesquiterpene hydrocarbons (18.01–28.58%), oxygenated sesquiterpenes (2.51–7.11%), diterpenoid hydrocarbons (0.19–0.39%), oxygenated diterpenoids (2.11–8.49%), and esters (0.69–1.03%). Among these classes, oxygenated monoterpenes represented the most abundant group. Overall, higher levels of oxygenated monoterpenes were detected in dried samples, with the maximum concentration occurring at the 1-hour HD. In dried samples, their concentrations progressively decreased with prolonged HD time. In fresh samples, although the highest concentration also occurred at the 1-hour HD, no marked temporal variation was observed thereafter. In fresh samples, the concentration of sesquiterpene hydrocarbons increased with HD time, except at the 5-hour treatment. In both fresh and dried samples, monoterpene hydrocarbons reached their peak concentration at the 1-hour HD. Subsequently, their concentrations decreased in fresh samples with prolonged HD time. Similarly, in dried samples, lower concentrations were detected at the 2-, 3-, 4-, and 5-hour treatments compared with the 1-hour HD. Diterpenoid hydrocarbons were absent during the first two HD periods of the dried samples. The concentration of oxygenated diterpenoids increased progressively with extended HD duration (Table 6). These results highlight the predominance of oxygenated monoterpenes and show that HD time and sample condition (fresh vs. dried) can shape the chemical profile of *S. fruticosa* VO under the applied conditions. Any implications of these compositional shifts for bioactivity would require dedicated biological assays.

The changes observed in the VO composition of *S. officinalis*—including the reduction of α-thujone and 1,8-cineole, the exclusive presence of nerol during the first hour, the absence of trans-β-farnesene, myrtenyl acetate, and epimanool in the initial fractions, and the increase in viridiflorol levels at later stages—are likely attributable to several interconnected factors. These factors may involve the differential extraction of volatiles according to their boiling points and volatility, together with the thermal decomposition and evaporation of certain constituents during extended HD. The observed variations in VO composition during HD can largely be attributed to differences in compound volatilities and their specific responses to thermal conditions. The VO obtained from *S. officinalis* leaves is typically characterized by elevated levels of thujone. In addition to thujone, the major constituents are 1,8-cineole and camphor^29^. According to previous studies, the decline in α-thujone content during HD can be ascribed to multiple mechanisms: (i) thermal or oxidative degradation due to extended exposure to heat and oxygen; (ii) fractionation and extraction kinetics, leading to a relative decrease of the compound as other constituents are recovered; (iii) derivative formation via hydrolytic or other chemical transformations; and (iv) retention or re-adsorption within the plant matrix^30–32^. Monoterpenes, owing to their low boiling points, tend to elute rapidly in the early stages of HD. Their relative abundance decreases over time, and they may also undergo thermal or oxidative degradation as a result of extended exposure. By contrast, oxygenated sesquiterpenes with higher molecular weights, such as viridiflorol, elute at later stages. This accounts for their detection at higher concentrations in the oil during extended HD periods.

Zheljazkov et al.^33^ reported that a short steam distillation duration of 1.25–5 min facilitated the recovery of camphor-rich VO from *Artemisia annua*, whereas extended distillation (up to 160 min) yielded higher concentrations of β-caryophyllene, trans-β-farnesene, and germacrene D. Furthermore, the partitioning of certain oxidized monoterpenes into the hydrolate phase, along with their retention or re-adsorption within the plant matrix, contributes to time-dependent variations in VO composition^30,33–36^. According to Handa^37^, the gradual changes in VO component concentrations during distillation are governed by each compound’s boiling point and water solubility. However, Sadeh et al.^38^ argued that the separation of VO constituents is determined more by their water solubility than by their boiling points. As the evaporation temperature remains constant throughout the process, changes in the relative proportions of the constituents can therefore be directly ascribed to the HD duration.

The higher α-pinene content observed in dried *S. fruticosa* samples is likely attributed to the reduced water content in the leaves following the drying process, which results in a higher concentration of compounds per unit of dry weight. The absence of diterpenes in short-term HD processes and their increased proportions with prolonged extraction can be attributed to their inherently low volatility. According to the European Medicines Agency^39^, the VO obtained from *S. fruticosa* leaves is primarily composed of oxygenated monoterpenes [1,8-cineole (40–67%), camphor (2–25%), and α- and β-thujone (less than 5%)], along with monoterpene and sesquiterpene hydrocarbons such as camphene, myrcene, α-pinene, β-pinene, and E-caryophyllene. Zheljazkov et al.^40^ reported that the concentrations of α-pinene and β-pinene were higher during short steam distillation periods (5–20 min), with levels decreasing as steam distillation time increased. The constituents of VOs exhibit varying vapor pressures and boiling points, which result in differential release rates from plant material. Accordingly, extending the HD process enables more efficient extraction of heavier and less volatile compounds^4^. However, it is important to note the potential downsides of prolonged distillation. Literature suggests that excessively long durations in different distillation methods can lead to an unpleasant odor in the VO. Conversely, a period that is too short in these methods may result in a deficiency of high-boiling components in the final product^25,41,42^.

### Chemometric analysis

**Fig. 1:**
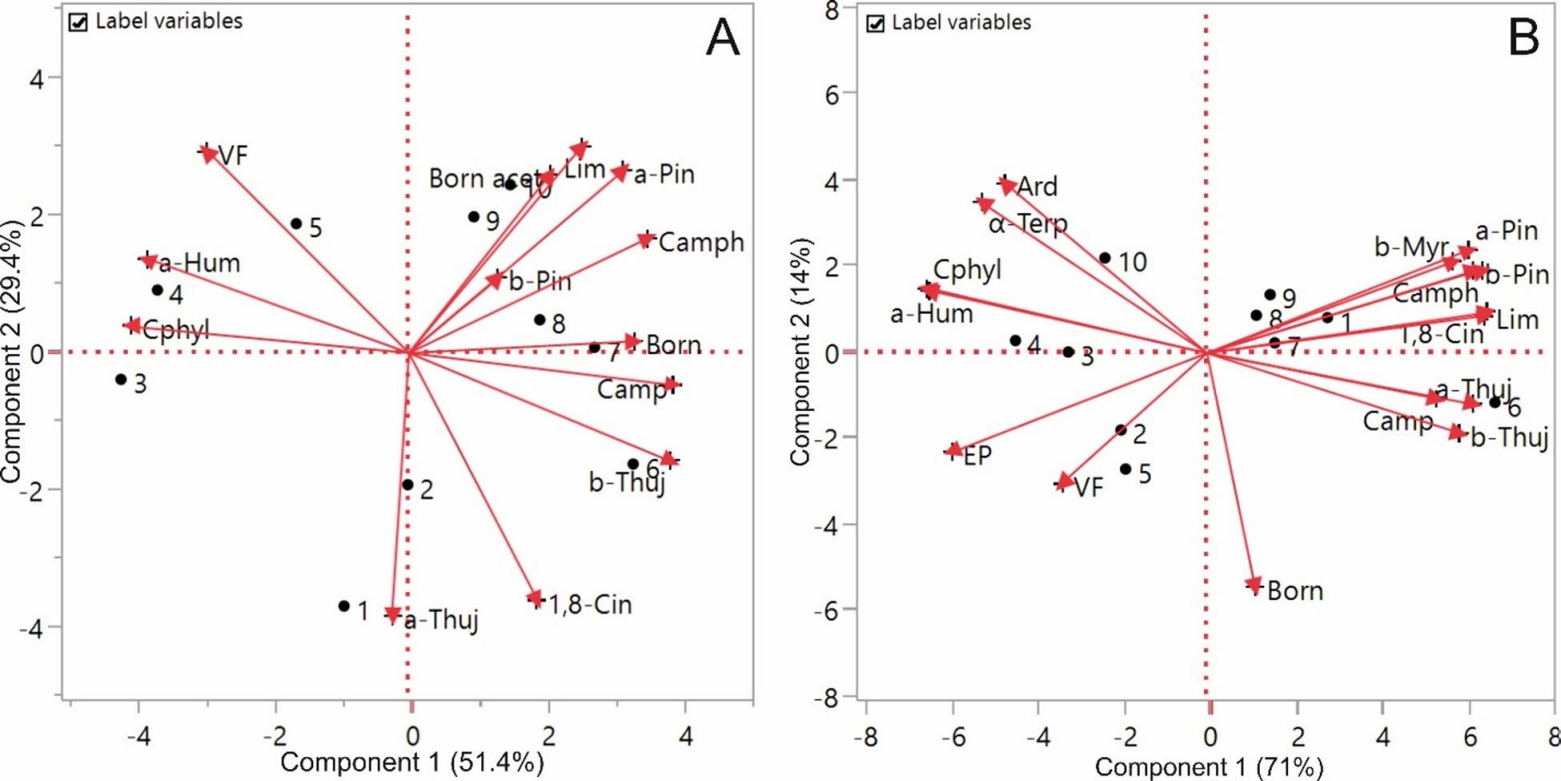
PCA biplots (PC1 vs. PC2) of the main volatile oil compounds in fresh and dried samples of *S. officinalis* (**A**) and *S. fruticosa* (**B**) by various hydrodistillation durations. Abbreviations for main VO compounds: 1,8-Cin: 1,8-Cineole; α-Terp: α-Terpineol; a-Hum: α-Humulene; a-Pin: α-Pinene; Ard: Aromadendrene; a-Thuj: α-Thujone; b-Myr: β-Myrcene; Born: Borneol; Born acet: Bornyl acetate; b-Pin: β-Pinene; b-Thuj: β-Thujone; Camp: Camphor; Camph: Camphene; Cphyl: Caryophyllene; EP: Epimanool; Lim: Limonene; VF: Viridiflorol. Abbreviations for HD durations: 1: Fresh-1 h; 2: Fresh-2 h; 3: Fresh-3 h; 4: Fresh-4 h; 5: Fresh-5 h; 6: Dry-1 h; 7: Dry-2 h; 8: Dry-3 h; 9: Dry-4 h; 10: Dry-5 h.

Based on the main VO components obtained from fresh and dried leaves of *S. officinalis* and *S. fruticosa* exposed to different HD durations, PCA was applied for chemometric classification and characterization of the samples, and the results are presented as biplots in Fig. 1.

For fresh and dried *S. officinalis* samples, the first principal component (PC1) was found to account for 51.4% of the total variance and the second principal component (PC2) for 29.4%, giving a cumulative explained variance of 80.8% (Fig. 1A). Examination of Fig. 1A showed that, in the characterization of fresh and dried *S. officinalis* samples, Dry-4 h (9) and Dry-5 h (10) were positioned in the upper right (positive) quadrant and were associated with bornyl acetate, limonene, α-pinene and β-pinene, while Dry-3 h (8) was related to camphene and Dry-2 h (7) to borneol and camphor. In the lower right (positive) quadrant, β-thujone was the main compound characterizing Dry-1 h (6). In the upper left (negative) quadrant, Fresh-5 h (5) was grouped with viridiflorol and Fresh-4 h (4) with α-humulene. In the lower left (negative) quadrant, Fresh-3 h (3) was characterized by caryophyllene, whereas Fresh-1 h (1) and Fresh-2 h (2) were both associated with α-thujone.

Along the PC1 axis, fresh samples were located predominantly in the negative region (left), whereas dried samples were located mainly in the positive region (right), indicating that leaf physical state was the dominant factor determining differences in the profile of major VO components. The orientations of the VO loading vectors supported this separation: the negative side of PC1 was aligned with sesquiterpenes such as viridiflorol, caryophyllene and α-humulene, whereas the positive side was associated with predominantly monoterpenoid compounds, including α-pinene, β-pinene, limonene, β-thujone, camphor and bornyl acetate. Samples of dried leaves exposed to longer HD durations (samples 7–10) were located closer to the vectors of bornyl acetate, limonene and α-pinene. In the fresh-leaf group (Fresh-1 h and Fresh-2 h), samples with short HD times were more strongly related to α-thujone, whereas samples obtained after longer HD times shifted towards the region associated with sesquiterpenes. Overall, these patterns indicated that, in *S. officinalis*, both drying and HD duration jointly shaped the balance between monoterpenes and sesquiterpenes in the VO.

For fresh and dried *S. fruticosa* samples, PC1 was found to explain 71% of the total variance and PC2 14%, giving a cumulative explained variance of 85% (Fig. 1B). As seen in Fig. 1B, in the characterization of fresh and dried *S. fruticosa* samples, Dry-4 h (9), Dry-3 h (8), Dry-2 h (7) and Fresh-1 h (1) were located in the upper right (positive) quadrant and were associated with β-myrcene, α-pinene, β-pinene, camphene, limonene and 1,8-cineole. In the lower right (positive) quadrant, Dry-1 h (6) was characterized by α-thujone, β-thujone and camphor. In the upper left (negative) quadrant, Dry-5 h (10) was separated mainly by aromadendrene and α-terpineol, while Fresh-4 h (4) was influenced by caryophyllene and α-humulene. In the lower left (negative) quadrant, Fresh-2 h (2) and Fresh-5 h (5) were classified primarily under the influence of viridiflorol and, to a lesser extent, epimanool.

Accordingly, the positive side of PC1 was aligned with vectors corresponding to α-pinene, β-pinene, β-myrcene, limonene and 1,8-cineole-compounds that are mostly monoterpenoid in nature-as well as with α-thujone and β-thujone, whereas the negative side of PC1 was co-oriented with heavier and relatively later-eluting constituents such as α-humulene, caryophyllene, viridiflorol, epimanool, aromadendrene and α-terpineol. This pattern showed that PC1 represented the main compositional balance axis, extending from a monoterpene-dominant profile towards a profile in which heavier, more slowly extracted constituents made an increasingly greater contribution.

In general, the PCA findings showed that the variation in VO compounds in both Salvia species is shaped by the combined influence of leaf condition (fresh vs. dry) and HD time. According to the study by Sellami et al.^17^ on *S. officinalis*, drying the plant, as compared with fresh material, induced significant changes in the abundance of oxygenated monoterpenes and major compounds such as 1,8-cineole, α/β-thujone, camphor and viridiflorol, indicating that the type and conditions of drying modify the balance between chemical classes. This pattern is consistent with the present findings for *S. officinalis*, as PCA likewise showed that the transition from fresh to dried leaves along PC1 shifts the profile from a sesquiterpene-associated pattern towards a predominantly monoterpene-rich profile (α/β-pinene, limonene, camphor, bornyl acetate). In the other study by Tibaldi et al.^18^ on three genotypes of *S. officinalis*, drying the inflorescences at 50 °C was reported to markedly alter the VO compound and to reduce both the oil content and the terpenoid fraction; the authors concluded that this temperature is not suitable for preserving terpenes and that milder conditions are preferable. These findings are comparable to the data obtained for *S. officinalis* in the present work, where the physical status of the leaves (fresh vs. dry) likewise did not act as a neutral pre-treatment but, together with the extraction conditions, systematically shifted the balance between monoterpenes and sesquiterpenes, a shift that is clearly reflected in the PCA biplot as the separation of fresh and dry samples along PC1. In a study on *Lavandula angustifolia* and *L. × intermedia*, Rathore and Kumar^58^ showed that the post-harvest drying duration (0–72 h) significantly affected the monoterpene/sesquiterpene ratio and the percentages of key constituents such as linalool and linalyl acetate. Prolonged drying led to directional changes in the contribution of several compounds, and the authors proposed that drying and/or extraction time should be adjusted according to the desired composition. This conclusion is in line with the pattern observed in both Salvia species in the present study, where PCA demonstrated that changes in pre-treatment conditions (fresh vs. dry) and process parameters can generate well-defined compositional gradients between monoterpene-dominated profiles and those enriched in heavier constituents.

In the study by Zheljazkov et al.^25^, the effect of steam distillation time (1.5–240 min) on the content and composition of *Lavandula angustifolia* VO was investigated. The authors concluded that distillation time can be used to “engineer” the chemical profile and to produce oils with different compositions from the same plant material. This pattern is comparable to the present results in that, in both Salvia species, PCA showed that different groups of constituents predominate at different time intervals; at shorter distillation times, volatile monoterpenes (such as α-thujone, α/β-pinene and limonene) are more abundant, whereas with prolonged HD heavier components (sesquiterpenes and diterpenoids) become more prominent in the final profile.

In the work of Jurevičiūtė et al.^59^ on *Thymus* × *citriodorus*, the effect of HD duration on VO compound was examined. Increasing HD time caused irregular changes in content, but a clear qualitative trend was observed: with longer times, the percentage of monoterpenes in the oil decreased, while the percentage of sesquiterpenes increased; the authors therefore concluded that HD longer than 60 min is not advantageous in terms of compound, particularly for preserving monoterpenes. These findings are in line with the data for *S. officinalis* and *S. fruticosa* in the present study, where the PCA biplots for both species showed that PC1 represents a gradient from a monoterpene-dominated profile towards one enriched in sesquiterpenes and other heavier constituents; notably, samples obtained after longer HD times were located in the region aligned with sesquiterpenes (such as viridiflorol, caryophyllene and α-humulene) and later-eluting compounds. In the study by Miguel et al.^16^ on *S. officinalis* VO obtained from dried aerial parts, the effect of different HD times on chemical composition was assessed. 1,8-Cineole, α-pinene and camphor were reported as the major constituents in all samples, and the authors concluded that, within the time range investigated, HD time did not have a marked effect on the relative percentages of the main components. This result differs from our findings in that, in the present study, PCA revealed that, although temporal changes in *S. officinalis* are secondary to the fresh/dry factor, they are still evident as a shift of samples between regions associated with monoterpenes and sesquiterpenes. On the other hand, both studies converge on the notion that the choice of extraction time can influence not only biological activity but, depending on plant material status and experimental design, also the profile of major constituents.

## Conclusions

This study indicated that HD duration and the initial state of the plant material (fresh vs. dried) were associated with differences in VO content and major-constituent profiles of *Salvia officinalis* and *Salvia fruticosa*. In *S. officinalis*, VO content increased with longer HD times, and dried leaves—particularly within the initial three hours—produced substantially higher content than fresh material, with diminishing returns thereafter, suggesting that ~ 3 h may be a practically sufficient duration for dried leaves under the applied conditions, whereas longer times may be selected when the aim is to maximize overall recovery. In *S. fruticosa*, although dried leaves yielded more oil on a dry weight basis, fresh leaves were preferable when considering VO content relative to the original fresh weight. GC/FID-MS profiling indicated that α-thujone was the predominant constituent in both species, with the highest relative abundance observed at 1 h, while other major compounds, including 1,8-cineole and camphor, decreased with prolonged HD. Oxygenated monoterpenes were the dominant chemical class, but their relative proportion declined over time, whereas sesquiterpenes and diterpenoids tended to increase during extended HD. These findings highlight species-specific extraction dynamics and underscore the importance of selecting HD duration to balance VO content with compositional characteristics. Overall, the results describe time-dependent compositional trajectories under the applied conditions and may inform process-time selection; however, confirmation with fully replicated extractions and analytical measurements is required.

## Data Availability

The author confirms that all data supporting the findings of this study are included within the article.
